# Immunotherapy as a treatment for type 1 diabetes mellitus in children and young adults: A comprehensive systematic review and meta-analysis

**DOI:** 10.1371/journal.pone.0321727

**Published:** 2025-04-11

**Authors:** Gaelle Salame, Vincent Hakim, Clara Dagher, Rose-Mary Daou, Anthony El Dada, Lea Nassif, Hilda E. Ghadieh, Sami Azar, Samer Bazzi, Frederic Harb

**Affiliations:** 1 Faculty of Medicine and Medical Sciences, University of Balamand, Kalhat, Lebanon; 2 AUB Diabetes, American University of Beirut, Beirut, Lebanon; Children's Hospital Boston, UNITED STATES OF AMERICA

## Abstract

**Background and Objective:**

Type 1 diabetes mellitus (T1DM) is characterized by the loss of pancreatic cells, resulting in total insulin insufficiency. According to the Diabetes Control and Complications Trial, T1DM treatment aims to achieve appropriate glycemic control and to prevent and avoid repeated episodes of hypoglycemia. Insulin therapy alone addresses the symptoms of the disease but fails to target the underlying pathophysiology of T1DM in children despite continuous efforts to enhance insulin regimens. Therefore, immunotherapy-based approaches have been considered potential treatments for T1DM in children since they can regulate the autoimmune responses and enhance the children’s quality of life by reducing their daily dose intake of insulin.

**Methods:**

In this meta-analysis, we have covered a few immunotherapeutic options based on preclinical and clinical data, namely, Teplizumab, Golimumab, Imatinib, Etanercept, Canakinumab, Ladarixin, Ala-Ala, Anakinra, and Otelixizumab in reliable databases such as Pubmed, Google Scholar, and Cochrane. SPSS was used for statistical analysis. Mean difference (MD) and standard mean difference (SMD) were used to evaluate the outcomes with a 95% confidence interval (CI).

**Results:**

To assess the effect of immunotherapy on the patients’ daily dosage of insulin and their HbA1c and C-peptide levels, data from twelve trials were combined and synthesized. Because of the high levels of heterogeneity in the selected studies, a random-effects model was used for analysis.

The combined data showed that patients receiving immunotherapy had higher C-peptide levels (Mean Difference (MD) = 1.51; 95% Confidence Interval (CI): [-2.56, 5.58]); however, this difference was not statistically significant (p = 0.42). On the other hand, patients in the immunotherapy group had significantly decreased HbA1c levels (MD = -0.63; 95% CI: [-1.18, -0.07]; p = 0.03), indicating that immunotherapy had a positive impact on glycemic management. Additionally, patients receiving immunotherapy exhibited a drop in their daily insulin dosage (MD = -1.15; 95% CI: [-2.59, 0.28]); however, this drop failed to achieve statistical significance (p = 0.10), thus indicating the need for additional research.

**Conclusion:**

This meta-analysis aimed to assess the effectiveness of immunotherapy in treating T1DM by examining its effects on the patients’ required dose of insulin, C-peptide, and HbA1c levels. While some studies failed to show desired results, the overall effect was an increase in C-peptide levels and a decrease in HbA1c levels. However, the study did not achieve statistical significance for insulin dosing. The main study’s strength is its focus on randomized clinical trials which is considered among the highest levels of epidemiological evidence. Therefore, further research is required to minimize the gaps and to explore immunotherapy-based drugs as potential treatments for T1DM.

## 1. Introduction

Type 1 diabetes mellitus (T1DM) is a chronic autoimmune disease driven by T-cells, leading to the gradual destruction of insulin-producing beta cells in the pancreas [1]. It often appears in children, but symptoms may take months or even years to become noticeable. Unlike prediabetes or type 2 diabetes (T2DM), the risk factors for T1DM are less well understood. Family history has been identified as a potential risk factor, as certain genes inherited from parents can increase susceptibility to T1DM [2]. However, many individuals with these genetic predispositions never develop the disease [2]. Additionally, environmental factors such as viral infections, dietary patterns, and exposure to specific chemicals may also play a role in triggering T1DM [2].

Diabetic ketoacidosis is more commonly observed in young individuals with T1DM, whereas adults with the condition are more likely to retain residual insulin production, as indicated by higher C-peptide levels [1,3]. Type 1 diabetes mellitus, accounting for 5% to 10% of all diabetes cases, has shown a steady rise in both incidence and prevalence worldwide [1]. A comprehensive review and meta-analysis reported an incidence of 15 cases per 100,000 individuals globally, with a prevalence of 9.5% [4]. The World Health Organization (WHO) has set a global target to halt the rise in diabetes and obesity by 2025, highlighting the urgent need for action. Currently, around 830 million people worldwide are affected by diabetes, with the majority living in low- and middle-income countries, and the disease is directly responsible for 1.5 million deaths annually [5]. The number of diabetes cases has consistently increased over the past few decades [5]. The standard treatment for pediatric patients with diabetes involves the use of exogenous insulin [6]. Advances in insulin delivery devices, glucose monitoring technologies, and the creation of new insulin formulations have greatly enhanced the quality of life and extended the life expectancy of individuals living with T1DM [6].

Exogenous insulin treatment controls blood glucose levels without targeting the underlying autoimmune mechanisms that lead to T1DM [6]. Studies have shown that immunotherapy treatments can alter the progression of T1DM [7]. Such immune-based strategies are generally classified as antigen-based and non-antigen-based immunotherapies [6]. Specifically, non-antigen-based immunotherapies encompass nonspecific and specific anti-inflammatory medications, treatments targeting T cells and B cells, and small molecules directed at alternative immune pathways. A considerable number of clinical trials that explored the aforementioned immunotherapies have achieved their primary outcome, which relied on C-peptide measurement [8]. Cyclosporine, an immunosuppressive drug, was initially studied as a treatment for T1DM; however, its application in clinical settings was restricted because of its nephrotoxicity and increased chances of developing cancer [7]. The first Food and Drug Administration (FDA) approved medication for T1DM, teplizumab (anti-CD3 monoclonal antibody), is one of the increasingly emerging immunomodulators used to delay the onset of stage 3 T1DM in adults and pediatric patients (aged ≥ 8 years) with stage 2 of T1DM [9].

Clinical research is currently being conducted to treat recent-onset T1DM using T cell-directed immune therapies, such as teplizumab, low-dose anti-thymocyte globulin, and the fusion protein abatacept (cytotoxic T lymphocyte-associated antigen-4-immunoglobulin), as well as antigen-specific immune therapies, like IMCY-0098, which have demonstrated encouraging outcomes [9]. Furthermore, the evaluation of otelixizumab (anti-CD3 monoclonal antibody) in patients with a new onset of T1DM has revealed that a metabolic response was observed at the 9 mg dose. In comparison, doses exceeding 18 mg were associated with an increased risk of EBV reactivation [10]. Unlike teplizumab, hematopoietic stem cells (HSCs) have immunomodulatory properties allowing them to have a strong immunoregulatory effect without reducing T lymphocytes. “Healthy” HSCs are essential for central tolerance, according to several studies. Modified HSCs cannot perform their immunomodulatory functions in autoimmune diseases such as type 1 diabetes. In mouse models, diabetes development has successfully delayed the onset of disease by addressing this deficiency. Although genetic engineering and ex vivo manipulation of HSCs have shown to be a successful procedure in mice, more research is required to determine their safety in humans [11].

Some observational studies have demonstrated the role of hematopoietic stem cells (HSC) and very small embryonic-like stem cells (VSEL) in maintaining the function of beta cell function and in partial remission of type 1 diabetes in children. A combination of a higher VSEL/HSC ratio and a lower HSC level is associated with a good predictor of a slowly progressive loss of beta cell function [12].

Stem cell therapy for diabetes has significantly progressed in the past decade, but challenges remain. MSC-derived IPCs are promising novel treatments for successful cell transplantation without encapsulation, immunosuppression, or genetic manipulations. They have insignificant teratogenic risk and can overcome autoimmune reactions in individuals with T1DM. However, their heterogeneity and donor-to-donor variability are the factors that delay their standardization protocols [13].

Therefore, innovative interventions such as immunotherapy-based drugs are beneficial to improve disease management and ameliorate the challenges faced previously in children with T1DM. However, the immunological effects, dose-response, safety, and tolerability should be monitored [10].

This meta-analysis discusses the rationale behind the necessity to explore novel insulin adjunct therapies such as immunotherapy-based drugs in the treatment of T1DM. It is important to note that early immunotherapy intervention is essential in the course of the disease to maintain beta cell function by promoting immune tolerance [8]. Immunotherapy drugs aim to reprogram the immune system to prevent the autoimmune destruction of pancreatic beta cells [8]. In addition, immunotherapy administration enhances the psycho-social lifestyle of children with T1DM by minimizing the dependence on exogenous insulin injections [8]. To provide a thorough assessment, this meta-analysis aims to measure the efficacy of different immunotherapies in pediatric T1DM patients by measuring their average C peptide levels, daily dosage of insulin, and HbA1c levels.

## 2. Methods

### 2.1. Eligibility criteria

In our meta-analysis, we defined eligibility criteria to ensure a focused and reliable synthesis of studies. Firstly, for inclusion criteria, we considered individuals aged 6–30 treated with antibodies and non-antibody treatments like tyrosine kinase inhibitors (Imatinib) or interleukin receptor antagonists (Ladarixin and Anakinra). The study population was limited to human subjects, and the primary outcomes measured were the area under the curve (AUC) of C-peptide and HbA1c levels in addition to the daily required dose of insulin, measured at least 6 months after immunotherapy treatment. Additionally, only studies using the Mixed Meal Tolerance Test (MMTT) were included. Exclusion criteria comprised any study not following a randomized control trial (RCT) design, eliminating follow-up studies, and those with AUC results not based on C-peptide, HbA1c, and insulin measurements.

NB: Guidelines used in sample meta-analysis:

1. Treatment Population: This category ensured that the age range, use of antibodies or non-antibody treatments, and human subjects’ criteria were met.
2. Intervention and Treatment Groups: Focused on the specific requirements of the immunotherapy treatment being studied.
3. Study Design: Ensured the selected studies adhered to RCT design, eliminating non-randomized or follow-up studies.
4. Outcome: Emphasized the significance of AUC results based on C-peptide measurements, highlighting the importance of the 6-month post-treatment interval and the use of MMTT.

### 2.2. Information Sources

We gathered data from various sources. Our primary searches were conducted in the Cochrane Library, PubMed, and Google Scholar, focusing on clinical trials. We specifically applied an RCT filter in PubMed to refine our results. Additionally, we accessed clinicaltrials.gov to ensure comprehensive coverage. The search in each database was last conducted in December 2023, and we excluded studies unrelated to our topic by refining keywords to guarantee relevance and precision.

### 2.3. Search Strategy

Our comprehensive search strategy aimed to capture a broad spectrum of studies on immunotherapy treatment for T1DM in children and young adults. We employed a set of tags and keywords and searched through relevant databases. The primary search terms included “Immunotherapy” AND “diabetes type 1” OR “juvenile diabetes,” exploring variations such as “Anti-CD3” AND “Diabetes type 1” and “Anti-CD3” AND “juvenile diabetes.” We also delved into specific aspects with searches like “Immunotherapy” AND “insulin-dependent diabetes” and refined specific inquiries like “Immunology AND treatment AND diabetes type 1 AND young adults” and “Immunology AND treatment AND diabetes type 1 AND children.” The use of these tags ensured a thorough exploration of the literature landscape while maintaining relevance to our study’s focus.

### 2.4. Selection process

**Fig. 1:**
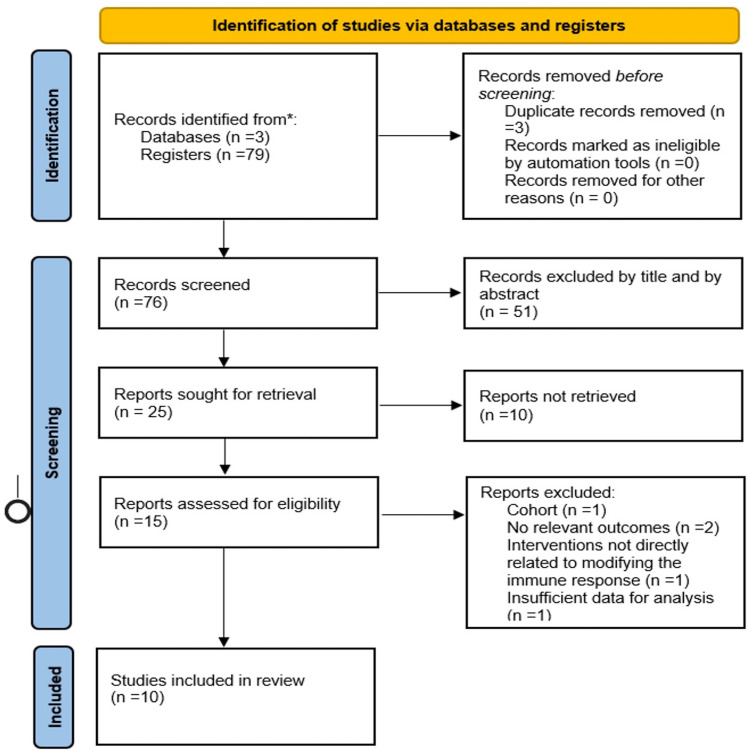
PRISMA flowchart of the included studies.

In our selection process, we followed the PRISMA flow diagram as represented in Fig 1. Initially, we screened the titles and abstracts of papers identified through our database search. Papers that aligned with our eligibility criteria or required a closer look for inclusion/exclusion decisions were shortlisted. Some studies respected our age criteria; however, some did not. We only considered the results of people ranging from 6 to 30 years old. For more information, please check the supporting information-article selection.xlsx.

To streamline data collection, we used a Google Doc form, where one author gathered information while another verified adherence to criteria.

The data collection involved extracting details like authors’ names, publication year, trial locations, outcomes, demographics, baseline insulin, and confirmation of ethical considerations. This thorough approach aimed to capture comprehensive information for our analysis. Six team members independently worked on the selection process, evenly dividing the workload to assess whether articles met the inclusion criteria. Notably, we opted for a manual review without automation tools, prioritizing a consistent evaluation to uphold the integrity of our study selection.

### 2.5. Data collection process

During the data collection phase, we carefully extracted crucial information from the selected reports. To enhance the reliability of our findings, each report was examined by two to three reviewers working independently — one reviewer took charge of data collection and the other ensured through cross-verification. This collaborative effort aimed to capture details beyond what was explicitly stated in the publications. Furthermore, we implemented a systematic process to obtain or confirm data directly from study investigators, fostering a deeper understanding of the trials and adding layers of authenticity to our datasets. This process involved direct communication via email (when needed), verification of supplementary materials provided in the included publications, and strict adherence to PRISMA guidelines (see SI1) to ensure accuracy and transparency in the data collection process. Importantly, our commitment to a manual review remained unwavering, and we abstained from using automation tools, prioritizing a human touch to ensure an accurate representation of the gathered information in our meta-analysis.

### 2.6. Data items

The primary outcome of our meta-analysis focused on the AUC of C-peptide and HbA1c levels and daily insulin dose dosage. We ensured that these values were consistently measured at the end of the treatment, predominantly after 6 months. This specific time-frame served as a critical reference point, aligning with our primary outcome assessment after the treatment period. We gathered results that were not only pertinent to our outcome domain but also standardized across studies in terms of measures, time points, and analyses. This approach aimed to enhance the comparability and coherence of our data, providing a good foundation for the synthesis of findings in our meta-analysis.

Beyond the primary outcome, our data collection included a comprehensive exploration of participant and intervention characteristics. We aimed to compare these characteristics over time, particularly concerning their baseline measurements. Additionally, we gathered information on funding sources for each study, thus recognizing the potential impact of financial support on study outcomes. In instances where information was missing or unclear, we made no assumptions and instead, prioritized transparency and accuracy. Our commitment to collecting and defining all relevant variables underscored the thoroughness of our approach and therefore ensuring that our meta-analysis was built on a foundation of well-defined and carefully gathered data points.

### 2.7. Study risk of bias assessment

In evaluating the validity of the included studies, we employed a risk of bias assessment for our meta-analysis. Two authors independently undertook the task of extracting data from each study to ensure a comprehensive and unbiased approach. In cases of any discrepancies, a thorough discussion was done to reach a consensus on the classification of risk of bias. To guide our assessment, we referred to the Cochrane Collaboration Risk of Bias tool (see SI2) whereby we examined various domains including selection bias, performance bias, and reporting bias. Each study was categorized into one of three classifications: low risk of bias, high risk of bias, or unclear risk of bias (see SI3). This process not only enhanced the transparency and reliability of our meta-analysis but also provided a good foundation for interpreting the validity and quality of the included studies in our systematic review.

### 2.8. Effect measures

In our study, we aimed to analyze continuous outcomes by calculating the mean difference (MD) and the standard mean difference (SMD, known as Cohen’s d in SPSS) of a successful outcome, here C-peptide levels, a marker of residual beta cell function in T1DM patients, after administration of specific immunotherapeutic agents for each trial in patients and control groups. Initially, the MD for each trial was calculated by subtracting the average C-peptide levels from the corresponding baseline measurements after the end of the exposure in each group. We did the same for HbA1C and insulin levels. In this way, we measured the average change in C-peptide as well as HbA1C and insulin levels due to the immunotherapeutic agent used in each trial. Second, SMD was computed whenever we had varying measurement units of C-peptide, HbA1C, and insulin levels among studies to ensure comparability.

### 2.9. Synthesis methods

First, we gathered the papers discussing the treatment of T1DM with teplizumab, ala-ala, and otelixizumab. Then, these papers were separated into human versus animal trials, filtering out the latter and keeping only human-trial articles to be used in our study.

The papers that made it through our inclusion criteria were separated based on the following characteristics: study design, interventions, age group of the sample size, and type of treatment used.

IBM SPSS Statistics 29.0.1.0 for Windows was used for statistical analysis. The model used was random effects to allow for heterogeneity and the incorporation of unobserved factors that can affect the values and therefore must be interpreted carefully. The method used was inverse variance. Also, the following methods were used to quantify heterogeneity: formal statistical test for heterogeneity, heterogeneity variance (T^2^), and inconsistency (e.g., I^2^). As for the variance estimator, we used Restricted Maximum Likelihood (REML). For the summary effect confidence interval, we used Truncated Hartung-Knapp-Sidik-Jonkman to explore the causes of statistical heterogeneity, and Egger’s regression base test was conducted. As part of the sensitivity analysis, the meta-analysis was conducted twice. The first run included all the initially chosen articles, and the second run only included the definitive articles, i.e., those that made it to the final results.

### 2.10. Reporting bias assessment

**Fig 2:**
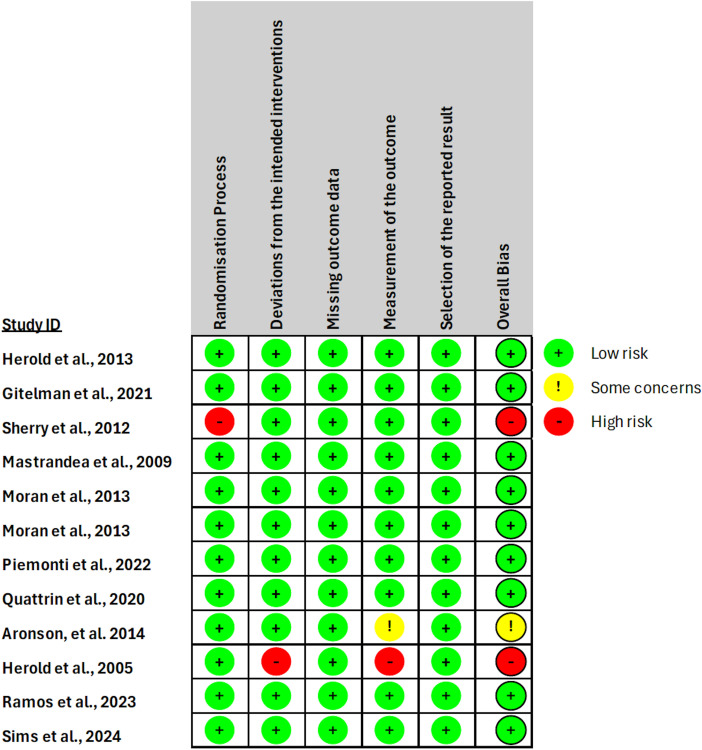
Traffic-light plot. Display of the risk of bias in the included studies based on Cochrane’s RoB 2.0 tool.

The selected articles were divided equally among six authors, who were then divided into groups of two. We used the Cochrane Collaboration’s risk of bias tool to perform our assessment. This tool evaluates the following sources of bias: random sequence generation, allocation concealment, blinding of participants and personnel, blinding of outcome assessment, incomplete outcome data, selective reporting, and other biases [14]. Each author within their assigned groups reviewed their assigned articles and conducted the assessment individually. Then, both authors in each group reviewed the assessment together to double-check that their answers matched. However, when the answers among authors differed, the writers within each group resolved the matter via discussion, each presenting their arguments as to why they chose that answer, and a consensus among both authors was ultimately reached. The tool contains five domains, each containing a set of questions to assess the overall bias in the article. The algorithm and assessor’s overall judgment concluded that only two of the twelve trials had a high risk of bias, and a third trial with “some concerns” was a biased assessment (more information are detailed in the supporting information-RoB analysis detailed.xlsx and Rob analysis-summary.xlsx). As for the rest, they were assigned a low risk of bias. The bias analysis results are summarized in Fig 2.

### 2.11. Certainty assessment

To assess the certainty of our body of evidence, we used the Grading of Recommendations Assessment, Development, and Evaluation (GRADE) approach:

- Risk of bias: by adhering to the Cochrane Collaboration Risk of Bias tool, and using Egger’s Regression-Based-Test, we had a high confidence interval, which made us confident that the true effect size fell within this interval.
- Heterogeneity: We found substantial heterogeneity across studies using the homogeneity/heterogeneity forest plot in SPSS for C-peptide levels (tau-squared: 30.952, H-squared: 499.650, I-squared: 99.8%). We found significant heterogeneity across studies for HbA1c levels (tau-squared: 0.514, H-squared: 11.306, I-squared: 91.2). Finally, for insulin dose (tau-squared: 2.750, H-squared: 54.936, I-squared: 98.2), we found some heterogeneity across studies since most of them fell within our confidence interval in the forest plot.
- Imprecision: by applying the trim-and-fill feature in SPSS, we could assess the precision by studying the confidence interval and sample size. We adjusted our study number until getting approved by trim-and-fill which resulted in an upgrade in certainty.
- Publication Bias: our funnel plot analysis suggested a low potential of publication bias, guiding us to further upgrading of certainty.

### 2.12. Registration information

This meta-analysis is registered in the PROSPERO database under the registration ID (CRD42024520526) and can be found on the PROSPERO website (https://www.crd.york.ac.uk/prospero/)

## 3. Results

### 3.1. Study selection

The PRISMA 2020 flow diagram for new systematic reviews (**Fig 1**) indicates the included searches of databases and registers only

We excluded five studies from our review (NIHMS748134; DIAGNODE-2; NIHMS1517070; ISRCTN14274380; NIHMS902279), and we provided reasons for their exclusion in the Characteristics of Excluded Studies tables. The ISRCTN14274380 trial was excluded because it lacked significant data and was presented as a protocol that does not offer actual statistical information suitable for analysis. Another study with the registration number NIHMS748134 was a cohort study, which means that it needs to meet the inclusion criteria set for our evaluation. Furthermore, the NIHMS902279 and NIHMS1517070 trials were omitted because they failed to provide outcomes relevant to our research and presented data obtained one and seven years after the ABATE trial, respectively. The DIAGNODE-2 (NCT03345004) research was eliminated because the treatment did not directly regulate the immune response through antibodies.

### 3.2. Study characteristics

The characteristics of the included studies are summarized in Table 1, providing an overview of study design, sample size, type of drug and interventions.

**Table 1:** Characteristics of Included Studies.

ID	Author	Year	Study design	Region	Sample size (n)	Duration of diabetes	Number of autoantibodies	Baseline C-peptide	Immuno therapeutic agent
1	Sherry et al. [15]	2011	randomized, placebo-controlled trial	North America, Europe, Israel, and India	305	12 weeks or fewer at the time of enrollment	positive autoantibody titre against an islet-cell antigen (ICA-512/IA-2), glutamic acid decarboxylase (GAD 65), or insulin,	Detectable fasting or stimulated C-peptide	Teplizumab
2	Gitelman et al. [16]	2021	multicenter, randomized, double-blind, placebo-controlled trial	Australia, United States	67	less than 100 days at the time of enrollment	at least 1 autoantibody (micro-assayed insulin, GAD-65, ICA-512, ZnT8 or islet-cell autoantibodies	at least 0.2 nM/L	Imatinib
3	Herold et al. [17]	2013	open-label, randomized, controlled trial	United States	58	at least 4 but not more than 12 months before enrollment.	at least 1+ autoantibody (islet cell antibody [ICA], anti-GAD65 or anti-ICA512).	at least 0.2 nM/L	Teplizumab
4	Mastrandea et al. [18]	2009	pilot randomized, placebo-controlled, double-blind study	United States	18	4 weeks	GAD-65 and/or islet cell antibody positivity	mean of 0.1 nM/L	Etanercept
5	Moran et al. [19]	2013	two multicenter, randomized, double-blind, placebo-controlled trials	USA and Canada	69	Within 100 days	at least 1 (micro-assayed insulin, GAD-65, ICA-512, islet-cell)	at least 0.2 nM/L	Canakinumab
6	Moran et al. [19]	2013	two multicenter, randomized, double-blind, placebo-controlled trials	Europe	69	Within the past 12 weeks	1 (GAD-65)	at least 0.2 nM/L	Anakinra
7	Piemonti et al. [20]	2022	multicenter, randomized, double-blind, placebo-controlled trial	Europe (Italy, Germany and Belgium)	76	Within 100 days	At least 1 (anti-GAD [GADA], anti-insulin [IAA], anti-IA-2 [IA-2A] or anti-ZnT8 [ZnT8A	at least 0.2 nM/L	Ladarixin
8	Quattrin et al. [21]	2020	multicenter, placebo-controlled, double-blind, parallel-group trial	United States	84	Within 100 days	At least 1 (anti–GAD 65, anti–islet antigen 2, anti–zinc transporter 8, anti–islet-cell antigen, or anti-insulin)	at least 0.2 nM/L	Golimumab
9	Aronson et al. [22]	2014	multicenter, randomized, placebo-controlled trial	U.S., Canada, and Europe	224	Within 90 days	At least 1 GAD-65; tyrosine phosphatase- like protein; zinc transporter autoantibodies; or insulin)	At least 0.2 nM/L	Otelixizumab
10	Herold et al. [23]	2005	randomized, controlled, open-label study	United States	42	Within 6 weeks	At least 1 (anti-GAD65, anti-ICA51 or anti-insulin)	0.2 nM/L	Ala-Ala
11	Ramos et al. [24]	2023	multicenter, double-blind, randomized, placebo-controlled trial	United States, Canada and Europe	328	Within 6 weeks	At least 1 autoantibody, antibodies (against GAD, zinc transporter 8, insulin, islet cells, or islet antigen 2)	0.2 nM/L	Teplizumab
12	Sims et al. [25]	2024	Randomized, placebo-controlled trial	United States	68	Not mentioned	More than 2 autoantibodies (islet autoantibodies)	0.05 nM/L on average	Teplizumab

NB: GAD-65: glutamic acid decarboxylase-65, ICA-512: islet-cell antigen-512

### 3.3. Results of individual studies

The first study we used in our meta-analysis reported C-peptide levels after Teplizumab treatment [15] was analyzed using a random-effects model. A significant increase in C-peptide concentration was observed during the effect assessment. (MD = 0.08; 95% CI: [-0.16, 0.32]), although this difference was not statistically significant (p-value = 0.52) compared to the control group. For HbA1c, Teplizumab showed no significant difference (MD = 0, 95% CI: [-0.24, 0.24], p = 0.97). Teplizumab did not significantly affect insulin dose (MD = -0.1, 95% CI: [-0.34, 0.14], p = 0.42). The second study we used in our meta-analysis revealed the impact of Imatinib [16] on C-peptide levels, analyzed using a random-effects model. The overall effect estimate demonstrated a modest increase in C-peptide levels (MD = 0.88, 95% CI: 0.36–1.41), with a statistically significant difference observed (p < 0.001) compared to the control/placebo group. For HbA1c, Imatinib showed a significant decrease (MD = -0.84, 95% CI: [-1.37, -0.31], p = 0). Concerning insulin, Imatinib also did not significantly affect insulin use (MD = -0.19, 95% CI: [-0.70, 0.32], p = 0.47). For the third study [17], Teplizumab treatment was associated with a considerable decrease in C-peptide concentration (MD = 3.35, 95% CI: [-4.06, -2.65]). A statistically significant difference was observed at a p-value of less than 0.001 compared to the control group. HbA1c was not significantly affected by Teplizumab (MD = 0.24, 95% CI: [-0.28, 0.75], p = 0.37). In our meta-analysis, the fourth study analyzed the effect of Etanercept [18] on C-peptide levels using a random effects model. Overall effectiveness assessment showed a significant increase in C-peptide concentration (MD = 5.52, 95% CI: 3.59, 7.45) with a statistically significant difference (p < 0.001) compared to the control group. The decrease in HbA1c level for Etanercept was significant (MD = 45, 95% CI: [-2.49, -0.40], p = 0.01). Regarding daily insulin intake requirement, Etanercept significantly decreased insulin use (MD = -2.35, 95% CI: [-3.56, -1.14], p = 0). Analysis of the fifth study revealed a considerable decrease in C-peptide concentration following Canakinumab treatment [19], with an overall effect estimate of MD = -2.84 (95% CI: [-3.52, -2.15]). This difference was statistically significant (p < 0.001) compared to the control group. For HbA1c, Canakinumab showed no significant difference (MD = -0.9, 95% CI: [-1.43, -0.37], p = 0). Regarding insulin, canakinumab decreased insulin dose (MD = -0.57, 95% CI: [-1.09, -0.06], p = 0.03). The sixth study [19] investigated the impact of Anakinra on C-peptide levels, showing an increase in C-peptide concentration (MD = 16.07, 95% CI: 13.38, 18.76). The difference was statistically significant (p < 0.001) compared to the control/placebo group. For HbA1c, Anakinra showed a significant decrease (MD = -2.29, 95% CI: [-2.89, -1.68], p = 0). Data for Anakinra was not available on insulin dose. The results from the study about Ladarixin [20] demonstrated a significant increase in C-peptide concentration following treatment, with an overall effect estimate of MD = 2.07 (95% CI: 1.48–2.65). This difference was statistically significant (p < 0.001) compared to the control group. For HbA1c, Ladarixin showed no significant difference (MD = -0.34, 95% CI: [-0.84, 0.15], p = 0.18). Regarding insulin, Ladarixin did not significantly affect insulin intake (MD = -0.18, 95% CI: [-0.67, 0.30], p = 0.46). Interestingly, preclinical data suggest that the use of Ladarixin, a non-competitive, dual allosteric inhibitor of CXCL8 (IL-8) receptors (CXCR1 and CXCR2), may reduce the burden of diabetic kidney disease as the IL-8-CXCR1/2 axis contributes to diabetic kidney disease. Studies indicate that ladarixin can prevent high glucose-induced adverse effects on renal cells, highlighting its role in protecting against diabetic nephropathy [26]. The study about Golimumab [21] reported a modest increase in C-peptide concentration with treatment, as indicated by an overall effect estimate of MD = 0.48 (95% CI: [0.03, 0.94]). This difference was statistically significant (p = 0.04) compared to the control group. For HbA1c, Golimumab showed no difference (MD = -0.38, 95% CI: [-0.83, 0.08], p = 0.11). Golimumab significantly reduced insulin dose (MD = -5.06, 95% CI: [-5.95, -4.17], p = 0). Analysis of the Otelixizumab trial [22] showed a slight increase in C-peptide levels after treatment, effect estimate of MD = 0.62 (95% CI: [0.37, 0.88]). This difference was statistically significant (p < 0.001) compared to the control group. For HbA1c, Otelixizumab did not show a significant difference (MD = 0.16, 95% CI: [-0.11, 0.42], p = 0.25). Regarding insulin, Otelixizumab did not significantly affect insulin dose (MD = -0.17, 95% CI: [-0.44, 0.10], p = 0.23). The tenth study demonstrated a significant decrease in C-peptide concentration following Ala-Ala treatment [23], with an overall effect estimate of MD = -3.55 (95% CI: [-4.51, -2.60]). This difference was statistically significant (p < 0.001) compared to the control group. For HbA1c, Ala-Ala showed a significant decrease (MD = -0.91, 95% CI: [-1.55, -0.27], p = 0.01). Ala-Ala significantly decreased insulin dose (MD = -0.96, 95% CI: [-1.60, -0.32], p = 0). The eleventh study focused on Teplizumab [24], which demonstrated a significant decrease in C-peptide concentration (MD = -0.47, 95% CI: [-0.70, -0.24], p < 0.001), indicating a reduction in pancreatic beta-cell function. However, Teplizumab did not significantly impact HbA1c levels (MD = 0.11, 95% CI: [-0.11, 0.34], p = 0.33). Additionally, there was a statistically significant decrease in insulin dose requirement (MD = -0.74, 95% CI: [-0.97, -0.50], p < 0.001), suggesting improved glycemic control or reduced insulin dependence. For the final study [25], also evaluating Teplizumab, a significant increase in C-peptide concentration was observed (MD = 0.66, 95% CI: [0.16, 1.17], p = 0.01), highlighting a preservation of beta-cell function. Furthermore, HbA1c levels showed a significant decrease (MD = -0.53, 95% CI: [-1.03, -0.03], p = 0.04), reflecting improved glycemic outcomes. Insulin dose data were unavailable in this study (MD = NA, 95% CI: NA, NA).

### 3.4. Results of syntheses

In nearly every study, the random order of allocating subjects was found to be unbiased, and therefore subjects were randomly assigned to the intervention groups. The order of the subjects was concealed until recruitment occurred to avoid selection bias. However, baseline differences between the two groups were not considered, causing questions about whether this randomization process was effective. At the beginning of the experiment, participants were unaware of their assigned interventions, which helped to reduce performance bias. The effects of the guidelines on the intervention were evaluated using a fit approach that improved the validity of the findings. The outcome data were available for all, or almost all, randomized individuals, which reduced the potential for attrition bias. The technique for evaluating the result was deemed suitable, and the assessment of the outcome between intervention groups was identical. Outcome assessors were unaware of the intervention proposed for study participants. A predetermined analysis strategy was followed when analyzing the data, which increased confidence in the results. No particulars were given regarding outcome measurement and analyses, causing concerns about biased reporting. Overall, the risk of bias for these papers was assessed to be minimal. However, for the study about Otelixizumab, Study ID 9 [22], data for the outcome were randomized for all participants, but there is evidence that the result might be biased due to data being available and unblinded to the monitors of the study. Also, for the study of Ala-Ala, Study ID 10 [23], participants were aware of their assigned interventions during the trial, and intervention deliverers were also likely aware of the assignments, indicating a significantly high risk of bias. Deviations from the intended intervention might have risen due to the trial context, and these deviations could have potentially affected the outcome. However, an appropriate analysis was used to estimate the effect of assignment on intervention, enhancing the validity of the findings. Also, for the study about Teplizumab, Study ID 3 [17], outcome assessors might have been aware or not of the intervention received by study participants, potentially influencing the assessment of the outcome. Whether this information could have influenced the outcome assessment is unclear. Although this study had several strengths, there was important participant and caregiver awareness of the interventions and potential deviations from the intended interventions that may have influenced the study results.

**Fig 3:**
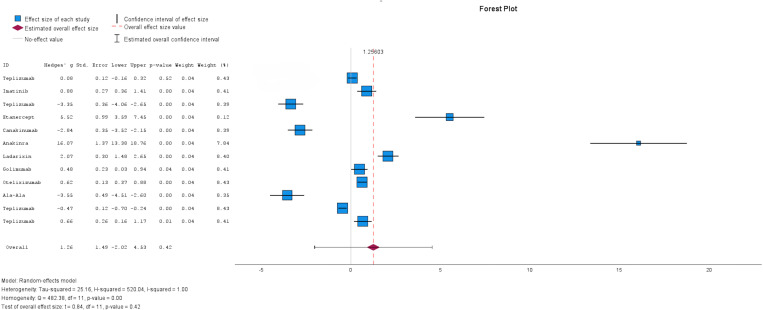
Forest plot of Mean difference of C-peptide levels in patients with Immunotherapy compared to the control or Placebo group. The size of the blue box indicates the study weight. The red diamond indicates the value for the total estimates.

The twelve studies reported the results of C-peptide levels (Fig 3), with high heterogeneity between studies (I^2^ = 100%), which were analyzed using a random-effects model. The overall effect estimate showed that patients receiving immunotherapy showed an increased C-peptide concentration (MD = 1.26, 95% CI: [-2.02, 4.53]). Yet, this difference was not statistically significant (p-value = 0.42) compared to the control/placebo group.

**Fig 4:**
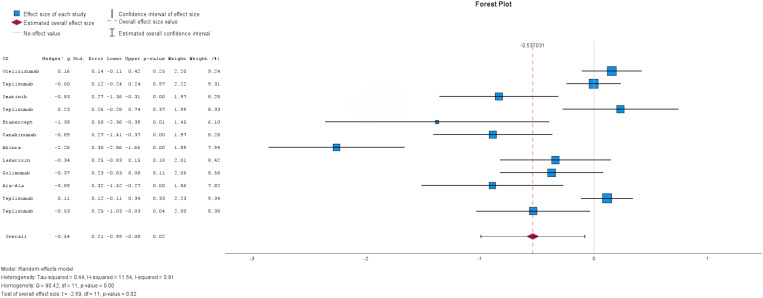
Forest plot of Mean difference of HbA1c levels in patients with Immunotherapy compared to Control or Placebo Group. The size of the blue box indicates the study weight. The red diamond indicates the value for the total estimates.

The twelve included studies provided data on HbA1c levels (Fig 4). There was considerable heterogeneity among the studies (I^2^ =91%), therefore a random-effects model was used for analysis. The overall effect estimate revealed a decrease in HbA1c levels among patients receiving immunotherapy compared to the control/placebo group (MD= -0.54, 95% CI: [-0.99, -0.08]). This difference was statistically significant (p-value = 0.02), indicating a beneficial effect of immunotherapy on HbA1c levels.

**Fig 5:**
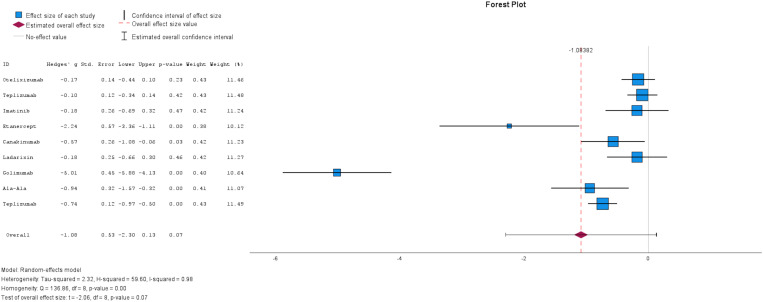
Forest plot of Mean difference of Insulin dose requirements in patients with Immunotherapy compared to Control or Placebo Group. The size of the blue box indicates the study weight. The red diamond indicates the value for the total estimates.

The findings from nine studies out of the twelve provided insights about insulin dose (Fig 5), revealing substantial heterogeneity among the studies (I^2^=98%). Consequently, a random-effects model was used for analysis. The overall effect estimate demonstrated a decrease in insulin dose among patients receiving immunotherapy, with a mean difference (MD) of -1.08 (95% CI: [-2.30, 0.13]). However, this difference did not reach statistical significance (p-value = 0.07), suggesting that the observed trend may not be conclusive. Further research is needed to elucidate the relationship between immunotherapy and the dose of insulin intake.

### 3.5. Reporting biases

Using the funnel plot and Egger’s regression test in our meta-analysis, we evaluated the presence of reporting biases. As suggested by the funnel plot asymmetry, we had potential publication bias, with smaller studies showing larger treatment effects. Egger’s regression test (with a 95% confidence interval of -0.414 & 6.785) confirmed this asymmetry, indicating a significant association between study size and effect size (0.13). These results imply that smaller studies with positive outcomes would be more likely to be published, which could cause our meta-analysis of treatment effects to be overestimated. To evaluate the risk of bias resulting from missing results in our meta-analysis, we employed the ROB tool. Regarding Domain 1, the adherence of each study to the randomization process was evaluated. Due to incomplete reporting, two trials (Study ID 3 and 10) raised questions about the randomization procedure. According to Domain 2 assessments, one study (Study ID 10) showed deviations from the intended interventions, which were highlighted as potentially influencing the outcomes. Domain 3 evaluations demonstrated that all studies treated missing outcome data appropriately and that no concerns popped up. On the other hand, evaluations of Domain 4 showed that studies varied in how outcomes were measured, and Study ID 9 raised concerns because of its uneven assessment methods. Concerns about Study ID 10’s selective result reporting—which could add bias to the meta-analysis—were noted in Domain 5. Finally, the majority of studies showed a low to moderate risk of bias in each of the individual domains; however, Study ID 10 raised concerns about overall bias because of selective reporting of outcomes and variations from targeted interventions.

### 3.6. Certainty of assessment

We assessed the certainty of evidence using the GRADE approach for each outcome included in our meta-analysis. The evidence was graded as high, moderate, low, or very low based on factors such as study limitations, inconsistency, indirectness, imprecision, and publication bias. For the primary outcome of C-peptide levels before and after immunotherapy, we assigned a grade of moderate due to the presence of a low risk of bias in the included studies and some inconsistency in effect estimates. Secondary outcomes including HbA1C, and insulin were generally graded as moderate to low due to limitations in study design, inconsistency, and imprecision of effect estimates. Based on the evaluation, it can be concluded that the findings of this meta-analysis are robust; however, the observed discrepancies in data are attributable to variations in the study designs of the included references rather than to the statistical methodologies employed in our analysis.

## 4. Discussion

This meta-analysis reviewed and analyzed the available literature on the effects of different novel immunotherapies on C-peptide and HbA1c levels as well as on insulin dose requirements in children and young adults with T1DM. The analysis was performed following the PRISMA 2020 guidelines.

In their paper, Ben Nasr et al., summarized the available literature on all immunotherapy-based clinical trials performed on patients with new-onset T1DM. However, their paper noted that most immunotherapies failed to establish insulin independence [27]. Hence, given the controversy around the efficacy of immunotherapy in diabetics compared to classical treatments, this meta-analysis was conducted to assess the effects of this type of treatment on obtaining the desired changes in C-peptide and HbA1c levels and insulin dose in individuals suffering from T1DM. Although some selected studies failed to show the desired results, the overall effect estimated a rise in C-peptide levels and a decrease in HbA1c levels with immunotherapy. However, there was a failure to achieve statistical significance for the former only, indicated by the resulting p-value. As for the effects of immunotherapy on insulin dose requirements, further research is still required. The results from our meta-analysis concerning the effect of immunotherapy on insulin dose were inconclusive.

There are multiple individual studies conducted on immunotherapeutic agents and their effects on our variables of interest. However, the meta-analyses were scarce. One meta-analysis published in 2024 [28] analyzed the effects of disease-modifying immunotherapy on C-peptide and HBA1c levels along with daily insulin dosage, and the risk of hypoglycemia. However, this meta-analysis failed to show any improvement in HbA1c but found a significant decrease in the daily required insulin dosage compared with placebo/controls [28]. The scarcity of meta-analyses, in addition to the conflict in results with our meta-analysis, emphasizes the need for further research on immunotherapy in the treatment of T1DM.

Our study possesses many strengths as well as some limitations. Starting with its strengths, our meta-analysis is one of the few meta-analyses discussing the implications of immunotherapy in treating T1DM. Additionally, our meta-analysis consists mainly of RCTs, which are of the highest level of epidemiological evidence. As for its limitations, our study failed to account for some underlying factors that might influence the clinical course of T1DM, such as the role of Bregs. The levels of Bregs, which is normally decreased in autoimmune conditions, positively correlated with fasting C-peptide and negatively correlated with hemoglobin A_1c_. On that note, Ben Nasr et al. noted that rituximab (B-cell depleter) can lead to exacerbations or de novo manifestations of autoimmune disease [29]. Additionally, there was variability in the size and composition of placebo groups across the included studies, such as differences in age, diabetes duration, and baseline C-peptide levels. This heterogeneity in placebo group characteristics could potentially influence the outcomes and reduce the precision of the pooled estimates in our meta-analysis. To mitigate these issues, we used a random-effects model, which accounts for between-study variability. However, we acknowledge that differences in placebo group composition remain a limitation, as they may have contributed to residual confounding that was not fully adjusted for in our analysis. Furthermore, there was variability in the length of follow-up periods across the included studies, ranging from 6 months to 2 years. This inconsistency in follow-up durations may have affected the comparability of reported outcomes, particularly those that require longer observation to assess their full impact, such as C-peptide preservation and insulin requirements.

Therefore, our study establishes a foundation for further research on the use of immunotherapy in combating T1DM, as it has identified several gaps that warrant exploration. Although satisfactory results were achieved concerning HbA1c and C-peptide levels, and daily insulin dosage requirements, the study was unable to conclude that immunotherapy improves the two latter outcomes. We are yet to reach a consensus that would recognize the use of immunotherapy as a standard treatment, similar to other approved daily treatments. In a nutshell, further research is still necessary.

## Supporting information

S1 File Supporting Information -Article selection. Table of Article Selection: A detailed table outlining the selection process, including the total number of articles screened, reasons for exclusions, and the final studies included in the analysis, following the PRISMA guidelines.
(XLSX)

S2 File Supporting information-RoB analysis-detailed table. Risk of Bias (RoB) Analysis: A comprehensive evaluation of the potential biases across studies, assessed using Cochrane RoB 2 tool. The detailed methodology, criteria, and justifications for each judgment are provided to ensure transparency.
(XLSX)

S3 File Supporting information-RoB analysis-summary table. Summary of the RoB Analysis: A concise summary presenting the overall bias levels (low, moderate, or high) for each study, along with visual representations.
(XLSX)

S4 File PRISMA_2020_checklist-Plos One. (PDF)
